# Head-to-head comparison of health-state values derived by a probabilistic choice model and scores on a visual analogue scale

**DOI:** 10.1007/s10198-016-0841-y

**Published:** 2016-11-02

**Authors:** Paul F. M. Krabbe, Elly A. Stolk, Nancy J. Devlin, Feng Xie, Elise H. Quik, A. Simon Pickard

**Affiliations:** 1 Department of Epidemiology, University Medical Center Groningen, University of Groningen, PO Box 30.001, 9700 RB Groningen, The Netherlands; 2 EuroQol Research Foundation, Marten Meesweg 107, 3068 AV Rotterdam, The Netherlands; 3 Office of Health Economics, 105 Victoria Street, London, SW1E 6QT UK; 4 Department of Clinical Epidemiology and Biostatistics, H306 Martha Wing, St. Joseph’s Healthcare Hamilton, McMaster University, 50 Charlton Avenue East Hamilton, Ontario, L8N 4A6 Canada; 5 Faculty of Mathematics and Natural Sciences, Groningen Research Institute of Pharmacy, University of Groningen, Antonius Deusinglaan 19713 AV Groningen, PO Box 30.001, 9700 RB Groningen, The Netherlands; 6 Outcomes and Policy College of Pharmacy, Department of Pharmacy Systems, University of Illinois at Chicago, 833 South Wood St, MC 886, Chicago, IL 60612 USA

**Keywords:** EQ-5D, Health states, Valuation methods, Discrete choice model, Visual analogue scale, I100

## Abstract

**Background:**

Health states were quantified based on discrete choice (DC) modeling and visual analogue scale (VAS) values using the five-level version of the EQ-5D (EQ-5D-5L). The aim of this study was to determine the extent of the relationship between DC derived values (indirect method) and VAS values (direct method).

**Methods:**

Data were collected in Canada, the United Kingdom, the Netherlands, and the United States. Respondents were asked to perform paired comparisons between two EQ-5D-5L health states for DC. In total, 400 different EQ-5D-5L states were included. After each DC task, respondents were prompted to score the two states one after another on a VAS. Intraclass correlation coefficients were calculated between DC and VAS values and illuminating graphs were designed.

**Results:**

Approximately 400 respondents participated from each country. High similarity [individual intraclass correlation coefficients (ICC) >0.85] of DC and moderate correspondence of VAS values were observed for the four countries. Cross-country comparison of DC values shows a nonlinear relationship to the VAS values.

**Conclusion:**

EQ-5D-5L derived DC and VAS values show a close but nonlinear relationship. Given the obvious biases associated with the VAS, DC methods based on ordinal responses may be a better alternative.

## Introduction

For decades, the merits and assumptions of direct methods of eliciting preferences for health states have been studied and debated. Direct valuation methods include the standard gamble (SG), time trade-off (TTO), rating scale, visual analogue scale (VAS), magnitude estimation, and person trade-off [1–3]. More recently, Thurstone scaling and extensions of this indirect (latent) method based on ordinal responses, namely the class of probabilistic choice models, have been explored in the field of health-status measurement [4–7]. All these valuation methods and latent measurement models are based on specific theoretical assumptions and arise from diverse disciplines. Empirical studies on the relationship between the values produced by each of these methods have revealed differences in the values elicited by the different methods and in their measurement properties [8]. So far, there is little agreement about which method is the most appropriate.

The use of different valuation methods has been of particular interest to developers of multi-attribute health classifier systems. The relationship between preferences generated by the various methods has been examined for the TTO, SG, and VAS [9, 10]. The relationship between discrete choice (DC) and TTO was recently examined by Pullenayegum and Xie [11], who found high correlations (*r* = 0.79 to 0.86) between health values measured through TTO tasks and latent values derived from DC data. Bijlenga et al. [12] investigated the agreement between values derived with DC and VAS values, and found a Cohen’s kappa of 0.79. To further understand the validity of the DC approach, this study examines the relationship between VAS- and DC-derived values and their underlying data structure using data from valuation studies for EQ-5D-5L conducted in four different countries.

## Theory

### Probabilistic choice models

Choice modeling offers an approach to explore people’s values, and has good prospects for health-state valuation. Actually, it relates to one of the oldest traditions in measuring subjective phenomena, namely the estimation of cardinal or metric measures based on ordinal responses. Thurstone’s ‘law of comparative judgment’ provides the conceptual foundation for most means of deriving cardinal values from ordinal assessments [13]. Following Thurstone, Bradley and Terry [14], Luce [15], and McFadden [16] further developed the basis for choice methods and refined their analytic capacity. Kind [16] presented the first application of the Bradley-Terry-Luce approach to health-state valuation.

What all probabilistic choice methods (logit or probit regression models) have in common is that they can establish the relative merit of one phenomenon (e.g., health states) with respect to others. Modern probabilistic choice models came from the field of econometrics and have been built upon the work of McFadden [17]. The models encompass a variety of experimental design methods, data collection protocols, and statistical procedures that can be used to predict the choices that subjects will make between alternatives (e.g., health states). These methods can be applied when subjects may choose between two or more distinct (‘discrete’) alternatives. In short, discrete choice models are grounded in modern measurement theory and are consistent with the random utility model in economic theory [18]. Interest in these methods has recently been revived in the area of health economics due to the relative simplicity of eliciting ordinal responses and the availability of a wide range of analytic tools to accommodate these responses [6, 19, 20].

### Visual analogue scale

Visual analogue scale (VAS), a renowned direct valuation method, originated in the social sciences and has been popular among psychologists. This type of scale has a long history and was initially called ‘graphic rating’ [21]. Aitken and Zealley were among the first to apply a VAS in medicine, using it to construct single-item mood scales [22, 23]. Ever since, the VAS has been a common research and clinical tool in psychological medicine, especially for measuring pain. Priestman and Baum [24] were probably the first to use a VAS (‘linear analogue self-assessment’) to assess quality of life among cancer patients. To our knowledge, Patrick and his colleagues were the first to use a VAS to derive values for hypothetical health states [25].

Essentially, a VAS (also sometimes called a ‘semantic differential’) is simply a straight line of a specified length with verbal descriptors at each end (anchors) consisting of short and easily understood phrases that describe the variable being measured. However, markers are often added to the line, usually with numbers attached. Formally called rating scales, these are often referred to as VAS, which for historical reasons is also the case for the EQ-VAS [26, 27].

The VAS is employed by developers of preference-based measures, such as the EuroQol Research Foundation, in several ways. The first is the conventional one in which an individual uses the VAS to indicate (e.g., to score or value) his own actual health status. Alternatively, the VAS is used to derive valuations of a set of hypothetical health states that are simultaneously assessed on one single VAS (multi-item VAS) such that a respondent evaluating outcomes A, B, and C must consider whether A is preferable to B, B preferable to C, and A preferable to C; the respondent also has to decide on the strength of these preferences.

### Measurement properties

There are theoretical and methodological differences between the direct valuation method (VAS) and the indirect (latent) measurement method based on choice models. Nonetheless, for both methods we assume that individuals have implicit preferences for health states, ranging from good to bad, and preferences can be revealed and expressed or derived quantitatively. Accordingly, differences between health states should reflect the increments of difference in the severity of these states. By implication, measures should lie on a continuous (unidimensional) scale. The differences between values would reflect true differences (e.g., if a patient’s score increases from 40 to 60, this increase is the same as from 70 to 90: interval level). This would mean that the values derived by different methods should have a linear relationship.

### Measurement mechanisms

With the VAS, the scores are directly positioned on a continuous scale (thermometer), and are equivalent to the values that we are interested in. As such, the VAS is a direct measurement method. Yet it should be kept in mind that in daily life people rarely make absolute judgments (i.e., attach a numeric measure, as done with the VAS). Most judgments consist of choices, and are thus inherently comparative. Therefore, the core activity of founded theories and models for subjective measurement, such as the DC models, is to compare two or more entities in such a way that the data will yield compelling information. Technically speaking, these models take individual values obtained at one measurement level and transform these to an aggregated level, specifically to produce an interval scale from ordinal data. So, response data produced by exercises as input for a choice model are not very informative. It is the inference based on the statistical algorithm of a specific choice model that produces the derived values. For this reason, such methods are referred to as indirect.

## Methods

### Study design

A study design was developed and implemented in Canada, England, The Netherlands, and the United States (US) between September 2010 and August 2011. Values for EQ-5D-5L health states were elicited by means of time trade-off (TTO; not presented), a choice model based on paired comparisons, and VAS. In this study, the most basic multi-item VAS was applied, whereby each time two hypothetical health states (from the paired comparison task) were scored one after the other.

### EuroQol-5D-5L

The health states selected for valuation were based on the EQ-5D-5L descriptive system. The EQ-5D-5L comprises the same five dimensions as the original three-level EQ-5D, i.e., Mobility (MO), Self-Care (SC), Usual Activities (UA), Pain/Discomfort (PD), and Anxiety/Depression (AD). In the EQ-5D-5L, however, the level structure is expanded, giving each dimension five levels: no problems, slight problems, moderate problems, severe problems, and extreme problems/unable to do something [28]. On the basis of responses to the EQ-5D health-state classifier, a preference-based scoring function can be applied that generates a single value for health.

### Respondents

In each of the four countries, at least 400 persons participated in the study. Representative samples from the general population (stratified by age, education, and sex) were recruited in each country with a minimum age of 18 years. Instructions and valuation tasks were presented and responses were collected within a digital setting by using a computer-assisted personal interview mode of administration: the EuroQol Valuation Technology [29]. To ensure that the valuation tasks were understood, the web-based assessments were interviewer-assisted. About three trained interviewers oversaw groups of approximately 15 respondents in six to eight sessions per day. In England, identical software was used; however, a team of eight home-based interviewers conducted the assessments in a one-to-one setting [30]. Respondents were paid a small sum by the panel administrators for completion of the survey. The exact amount, which differed across the countries, was in the range of €20 to €60.

### Experimental design

A Bayesian algorithm was used to generate an efficient design consisting of 200 paired comparisons (i.e., 400 health states). Priors in the estimation algorithm were based on an earlier study [31]. The 200 paired comparisons were subdivided into 20 blocks so that each respondent would make 10 paired comparisons.

### Response tasks

**Fig. 1:**
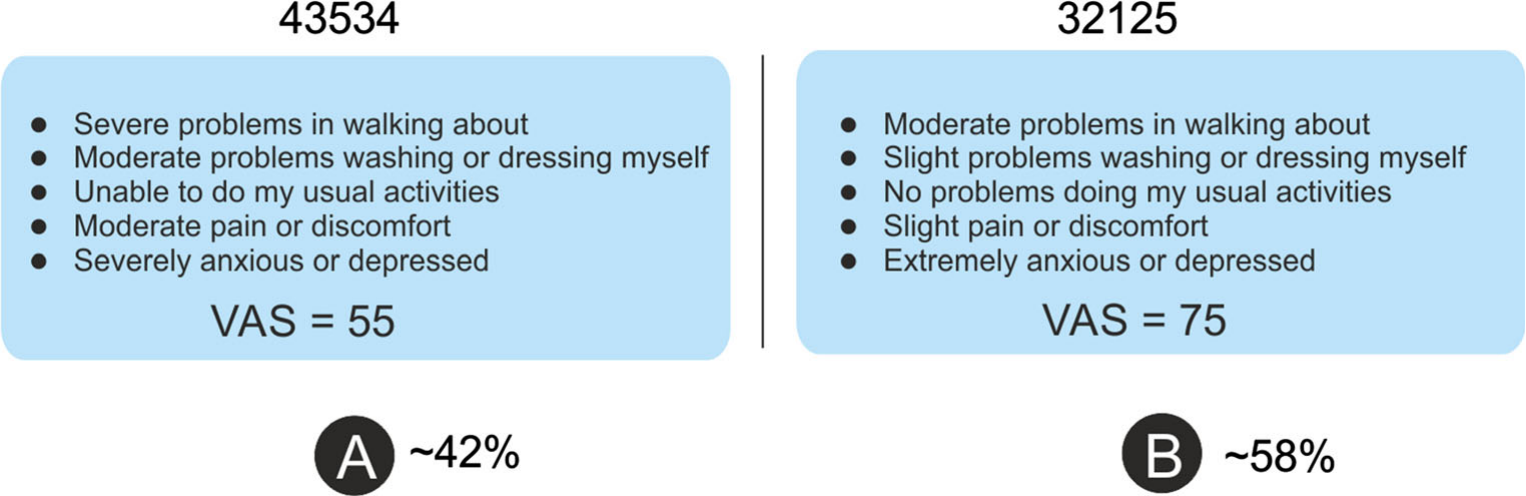
Example of the paired comparison task for the EQ-5D-5L pair 43534 vs. 32125 [also presented percentage of respondents choosing in the discrete choice (DC) task for state A or B, and the visual analogue scale (VAS) values for these two states]

Respondents were asked to perform the most simple response task in the framework of choice models, namely a paired comparison between two EQ-5D health states (Fig. 1). Everyone had to make a forced choice between ten different pairs of EQ-5D-5L states. These paired comparison tasks did not include “dead” or duration statements. No “status quo” or “opt-out” choices were offered. In total, 200 pairs of states (400 states in all) were judged (order of the pairs and order within each pair had been completely randomized by the computer system) in each country [32]. After each paired comparison (e.g., DC task), respondents were prompted to assess the two states on a single VAS. First, they assessed the state that had been judged as the best health state in the DC task; then they assessed the other health state, whereby the placement of the first one was shown on the VAS as well. At the start of the session with the paired comparisons, an animation popped up on the screen to explain the general purpose of the task and instruct the participant on what to do. This animation also explained the VAS tasks that proceed from the paired comparison tasks.

### Analysis

For the DC analysis, a multinomial probit model (alternative-specific multinomial probit, STATA) was used to analyze the paired comparison data. This is equivalent to standard approaches to analyzing paired comparison data or discrete choice data (e.g., McFadden model). The main-effects model included 20 dummy variables to represent each level 2, 3, 4, and 5 on each of the five domains: MO, SC, UA, PD, and AD. The values derived from this method are on an undefined scale (no meaningful anchors such as 0 = dead and 1 = full health). Expressed in a formula, the model predicts latent values or utilities ***v*** of individuals choosing health state *s*; *γ* represents a single vector of unknown regression coefficients; and *z*_rs_ indicates a vector of alternative-specific explanatory variables (e.g., dummies) for respondent* r*; *ε*_ij_ is an error term.

$$v_{\text{rs}} = \, \gamma z_{\text{rs}} + \, \varepsilon_{\text{ij}} \Rightarrow$$

Added to our model is an alternative-specific constant (ASC) capturing a tendency to always choose the first option, which makes the full formula:

$$\nu_{\text{rs}} = \gamma_{0} {\text{ASC}} + \gamma_{1} {\text{MO}}2 + \gamma_{2} {\text{MO}}3 + \gamma_{3} {\text{MO}}4 + \gamma_{4} {\text{MO}}5 + \gamma_{5} {\text{SC}}2 + \cdots + \gamma_{20} {\text{AD}}5 + \varepsilon_{\text{ij}}$$

The VAS values were aggregated to 400 EQ-5D-5L pooled states per country, whereas for the DC the 400 states presented in the paired comparison tasks were predicted by the formula. For VAS and DC, individual intraclass correlation coefficients (ICC) were calculated between values per country versus the pooled values of all four countries. Pearson’s correlations, ICCs, and quadratic regression functions were calculated between VAS and DC values. The DC values were regressed on the VAS values.

Charts for all combinations of countries and their regression functions were made in SigmaPlot (version 12.0; Systat Software, San Jose, CA) to investigate differences in constant and slope. Detailed graphs were also constructed to reveal the underlying distributions between DC and VAS values.

## Results

### Respondents

The number of individuals who entered the study was 547 for Canada, 404 for the UK, 407 for the Netherlands, and 417 for the US. A total of 1775 respondents completed all 17,750 paired comparisons. Age distribution was similar across the four countries, although the Netherlands had a smaller proportion of younger participants and a larger proportion of middle-aged ones (Table 1). The mean age in the entire dataset was 40 years (SD 16), with a range of 18 to 100. Regarding gender, the differences between countries were modest. The samples closely matched the populations on these key characteristics. Additional demographic information was collected only for the US and the UK. Among US respondents, 70.8% reported that they had received education beyond high school; 65.8% were non-Hispanic white (*n* = 273), 17.6% African American (*n* = 73), and 16.6% other ethnicities. The UK sample included a larger proportion of degree-educated and employed individuals compared to the general population, but the sample was broadly representative in terms of other background characteristics, such as ethnicity [33].

**Table 1:** Characteristics of participants from the four countries

	Canada (*N* = 547)	UK (*N* = 404)	The Netherlands (*N* = 407)	US (*N* = 417)
Male, % (*N*)	100 (230)	100 (202)	100 (198)	100 (211)
18–24	26.5 (61)	25.7 (52)	17.7 (35)	20.8 (44)
25–34	25.2 (58)	23.8 (48)	11.1 (22)	28.9 (61)
35–44	15.2 (35)	21.8 (44)	22.7 (45)	15.6 (33)
45–54	16.1 (37)	16.3 (33)	24.2 (48)	18.5 (39)
55–64	8.7 (20)	6.4 (13)	17.2 (34)	8.1 (17)
65–74	4.8 (11)	4.5 (9)	6.1 (12)	7.1 (15)
75+	3.5 (8)	1.3 (3)	1 (2)	1 (2)
Female, % (*N*)	100 (317)	100 (202)	100 (209)	100 (206)
18–24	21.4 (68)	25.8 (52)	12.0 (25)	17.5 (36)
25–34	20.2 (64)	31.2 (63)	17.2 (36)	19.4 (40)
35–44	13.9 (44)	15.8 (32)	27.3 (57)	14.6 (30)
45–54	16.1 (51)	15.4 (31)	26.7 (56)	21.8 (45)
55–64	15.5 (49)	5.9 (12)	14.4 (30)	18.9 (39)
65–74	9.8 (31)	4.5 (9)	1.9 (4)	5.3 (11)
75+	3.2 (10)	1.3 (3)	0.5 (1)	2.4 (5)
Age, Mean (SD)	40.3 (17.3)	36.4 (15.0)	42.2 (14.2)	40.4 (16.0)

### Completion

The number of judgments for each separate health state in the four countries ranged from 15 to 42 (mean 22.5, SD 2.68). In the Dutch study, one block of states (block 11) was not assessed due to a programming error. The number of drop-outs (individuals not completing all of the valuation tasks) was low in all countries (ranging from UK 4 to Netherlands 14). For the Netherlands and the UK, the average duration (seconds) per DC task was 32.5 and 45.2, respectively. For Canada it was 35.85 (SD 39.50, minimum 0.81, maximum 494.1), and for the US it was 29.16 (SD 37.07, minimum. 0.91, maximum 332.88). It took less time (22.71 s; SD 0.16) to complete a VAS than the paired comparisons (32.95 s; SD 0.28; *P* = .00).

### Health-state values

Pooled DC coefficients were all statistically significant. All showed a logical increase that corresponded with the underlying structure of the levels of the attributes (Table 2). One exceptional finding is that the coefficient of slight pain/discomfort (PD2) was more negative than that of moderate (PD3) pain/discomfort. The average DC values per country differed similarly to the average VAS values for the set of 400 EQ-5D-5L states, showing the highest mean values over all health states in the US, followed by Canada, then by the Netherlands, with the lowest values in the UK.

**Table 2:** Discrete choice parameter estimates (probit regression) based on responses from all countries

	*N* = 1775 (547 + 404 + 407 + 417)		
	Obs = 35,500 [1775(j)×10(pairs)×2(I)]^a^		
	Coef	SE	Sign
Constant^b^	−0.124	0.015	0.000
MO2	−0.299	0.031	0.000 0.000
MO3	−0.349	0.035	
MO4	−0.923	0.036	0.000
MO5	−1.326	0.039	0.000
SC2	−0.208	0.033	0.000
SC3	−0.290	0.035	0.000
SC4	−0.793	0.036	0.000
SC5	−0.966	0.035	0.000
UA2	−0.194	0.032	0.000
UA3	−0.254	0.035	0.000
UA4	−0.769	0.035	0.000
UA5	−0.987	0.035	0.000
PD2	−0.248	0.033	0.000
PD3	−0.241	0.035	0.000
PD4	−1.017	0.036	0.000
PD5	−1.258	0.036	0.000
AD2	−0.195	0.034	0.008
AD3	−0.454	0.035	0.000
AD4	−1.183	0.037	0.000
AD5	−1.401	0.038	0.000
Log likelihood	−9043.843		
Wald chi2 (20)	4817.43		
AIC	18,129.686		
BIC	18,307.709		
Degrees of freedom		21	

^a^ *I* Number of alternatives, *j* number of respondents

^b^In the set of coefficients the constant represents the alternative specific constant, capturing a tendency to always choose the first option

### Comparability of countries’ health-state values

**Fig. 2:**
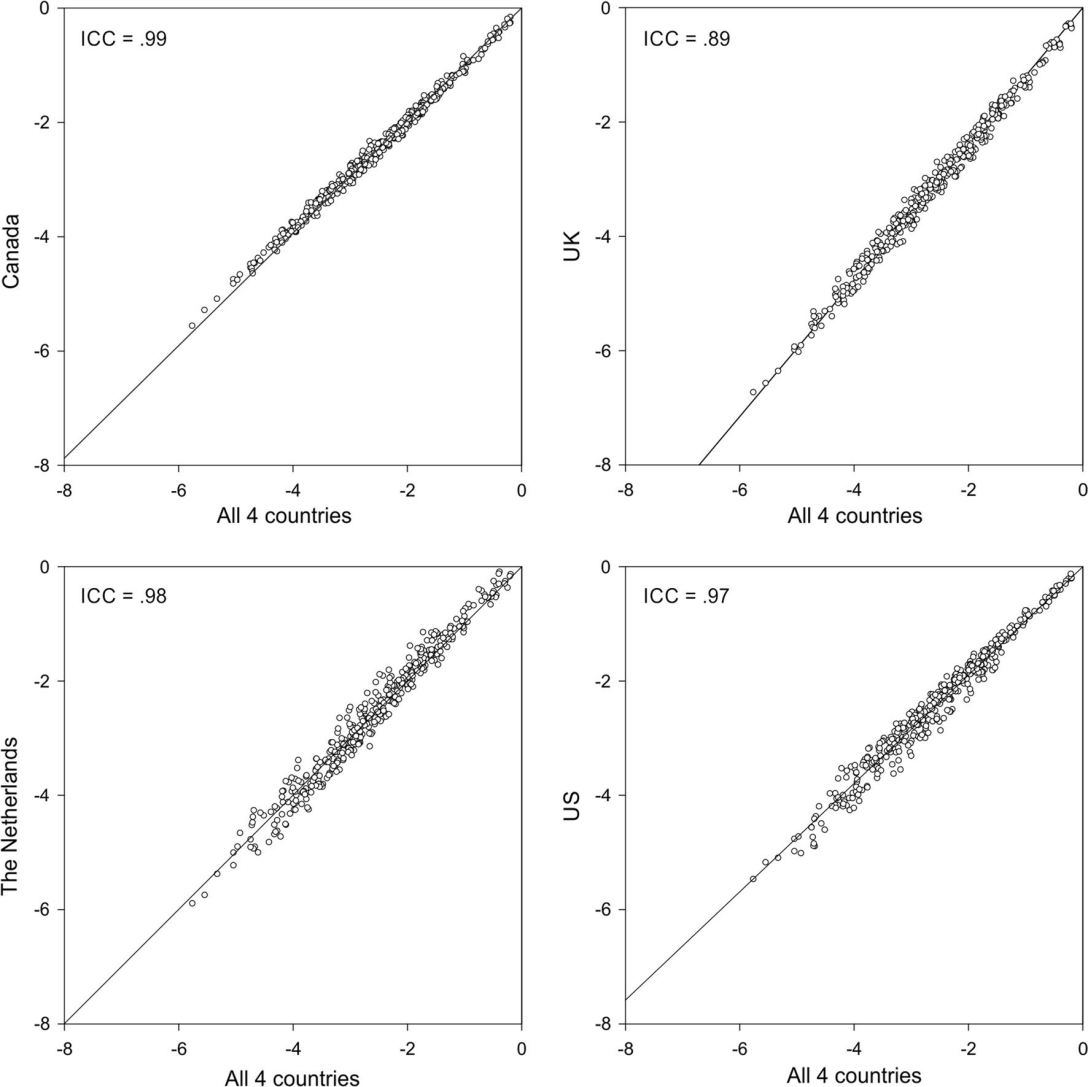
DC-derived values per country compared to the pooled DC value of 400 EQ-5D-5L states

**Fig. 3:**
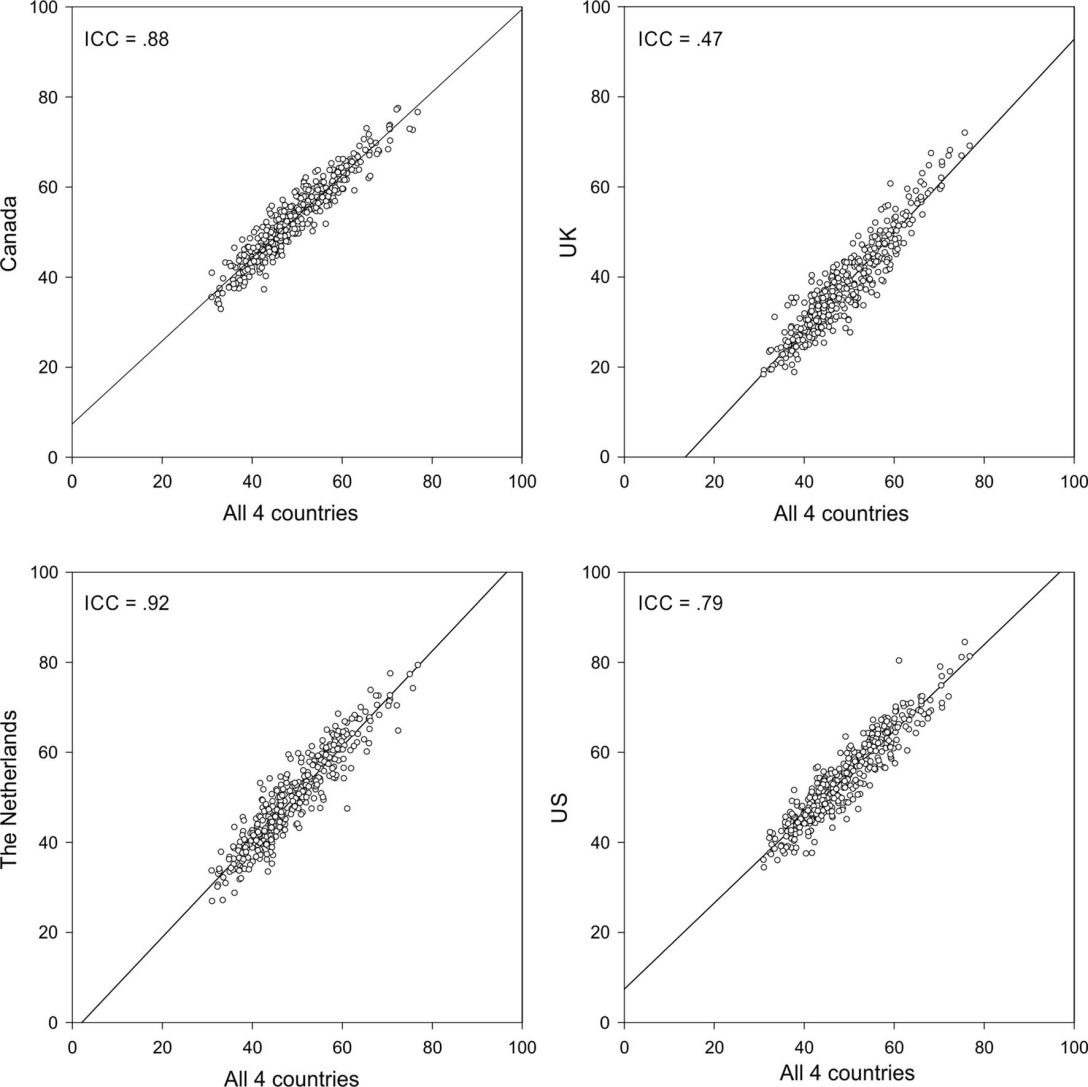
VAS values per country compared to the pooled VAS value of 400 EQ-5D-5L states (DC* scale*: 0 = best health state)

When the DC values for the 400 EQ-5D-5L health states are compared to the pooled DC value, the result is a high Pearson’s correlation: almost 1.00 for Canada, 0.99 for UK, 0.99 for the Netherlands, and 0.99 for US (Fig. 2). The VAS values for the 400 EQ-5D-5L health states showed strong correlations with the pooled VAS value: Canada 0.95, UK 0.93, the Netherlands 0.93, and US 0.93 (Fig. 3). The values for differences between severe health states are more equal with the VAS than under the DC model.

### Comparability of the two valuation methods

**Fig. 4:**
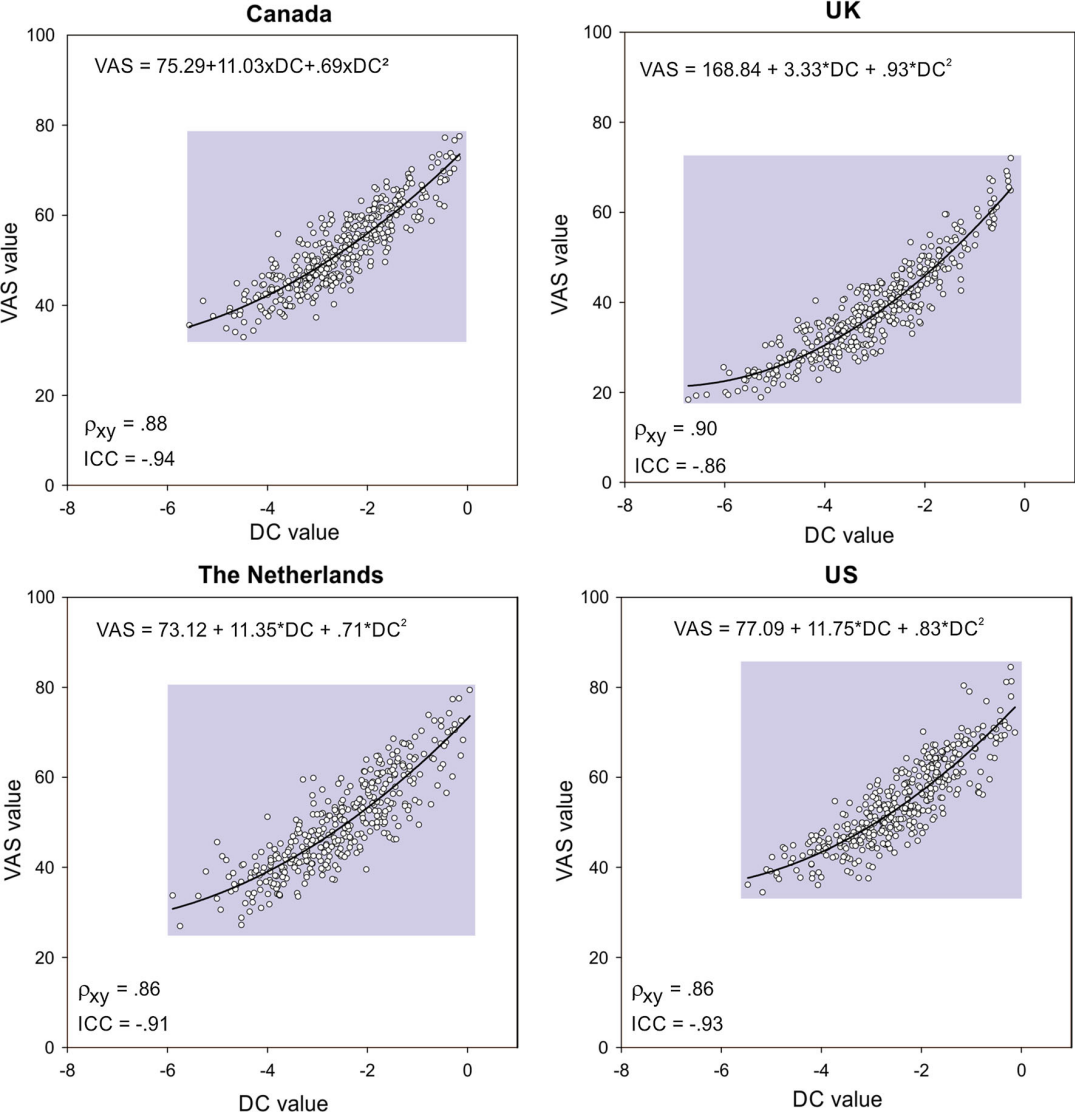
Comparison of VAS values and DC-derived values per country for 400 EQ-5D-5L states (*scale*: 0 = best health state)

The cross-country comparison of the pooled DC values and pooled VAS values of the 400 EQ-5D-5L health states showed a strong correlation (*r* = 0.93). Comparisons between the individual countries showed strong correlations: Canada 0.88, UK 0.90, the Netherlands 0.86, and US 0.86 (Fig. 4). The UK values for VAS as well as DC were found to be lower than the pooled values and the other three countries’ values.

**Fig. 5a–c:**
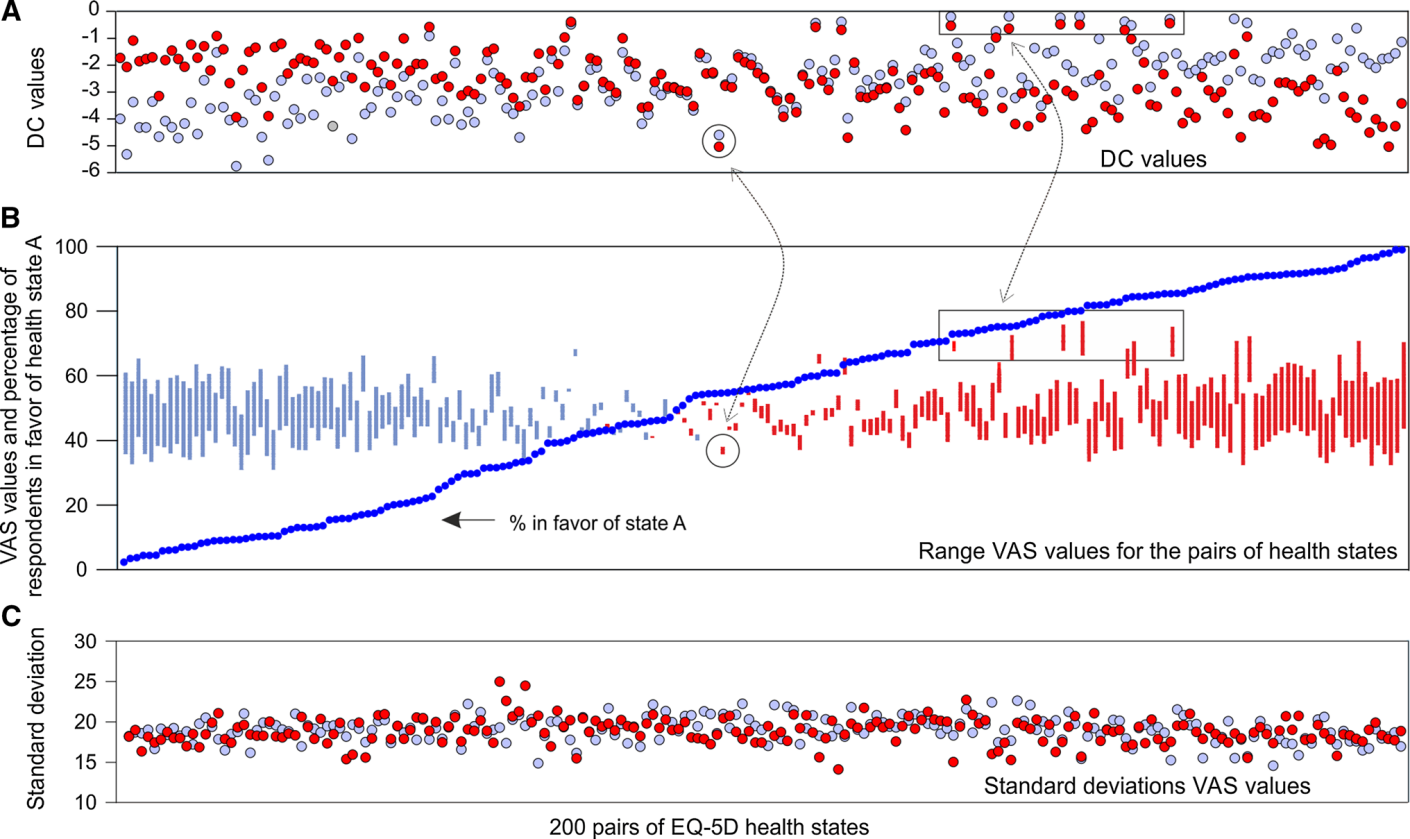
Descriptive representation of the 200 pairs of health states (*light blue* state* A*, *red* state* B*) for the derived values based on the DC model and the VAS.** a** Predicted DC values for respondents in favor of health state *A*.** b** Range VAS values for each pair (*light blue* higher value for state* B*, *red* higher value for state* A*).** c** Standard deviations VAS values. On all three graphs, the *x*-axis is ordered on percentage of respondents in favor of health state* A*

Graphical representation of the values obtained by the two methods in relationship to (the pairs of) states showed the following. First, mean VAS values were all positioned in a relatively small range (ca. 40–70) of the total scale (Fig. 5b). Small perceived differences between two states of a pair (e.g., close to 50% choice in favor of one of the two states in the DC task) are reflected in small differences between VAS values (Fig. 5b). Differences in values for the VAS pairs (Fig. 5b) are also clearly reflected in the derived DC values (Fig. 5a). Another correspondence between the DC and VAS was revealed: for the VAS, although respondents tend not to score on the boundaries (<30, >70), pairs of states that were scored low or high on the VAS were predicted under the DC model also as low or high. A clear example of this correspondence is depicted in Fig. 5a and b (see: two circles, two boxes). The standard deviations of the VAS values are relatively homogenous among the 200 pairs of health states that include pairs of rather modest states and pairs of rather severe states (Fig. 5c).

## Discussion

The cross-country comparison of DC and VAS values demonstrated a nonlinear relationship between the two methods. The curvature in the relationship suggests that values differ more at the ends of the scale. In this case, this implies that differences between worse health states are greater under the DC model, and differences between relatively good health states are larger in the VAS. Divergence between the four countries was modest, although the UK showed a small deviation as the values of VAS, and DC showed lower values for the worse health states. However, it is hard to say whether any differences in these values are due to cultural notions, methodological differences, or to translational issues (e.g., Dutch wording may make levels 2 and 3 seem closer together than in other language versions).

A limitation of the study is that the assumed independence of the VAS- and DC-derived values is reduced due to the chained nature of the task. Moreover, when the respondents positioned two health states simultaneously on a single scale (multi-item VAS), consistency with the preceding discrete choice task was required. They were offered the opportunity to redo that choice or correct their VAS score. In case two health states were compared that were very different, the chained nature would hardly affect both assessments; however, if the two states were relatively similar, differences in the VAS scores might be inflated. Overall, the multi-item VAS may be regarded as a compound task in which the prior comparison is supplemented with a level of rating, thereby enforcing congruence between methods.

The implication of the observed nonlinear relationship between DC and VAS is that the differences between health-state values obtained by one method are not proportional to the differences obtained by the other one. The curvature in the relation between DC and VAS can be partially explained by referring to theory and empirical evidence on VAS biases. Many studies have shown that the VAS is prone to diminished use of the upper and lower part of the scale (end-aversion). That propensity leads to range reduction and the typical curviness of VAS values as compared with other valuation methods [34]. This phenomenon is known from studies that compared VAS values with methods such as standard gamble and TTO [9, 10]. Any nonlinear relationship indicates that one, or even both, valuation methods are producing values that do not possess interval level or cardinal measurement properties. The limited value range of the VAS observed in this study seems to confirm this phenomenon.

We are aware of the longstanding theoretical debate about whether or not interval properties can be ascribed to VAS. Economists claim that responses to the standard gamble and the TTO have interval scale properties, whereas responses to rating scales, including the VAS, tend not to have interval scale properties because no trade-offs are expressed. Attempts have been made to find empirical evidence that mean health-state values collected with a (multi-item) VAS can be characterized roughly as interval data. One study, based on a rank-based scaling method (unfolding), observed a very strong relationship that supports the interval scale property of the VAS data [35]. Confirming results were found in a study that applied nonmetric multidimensional scaling on data (metric and ranks) that were derived from VAS values [36].

On the other hand, it is well documented that the VAS is prone not only to end-aversion but also to context or reference bias. The presumed independence of the set of health states to be positioned on the VAS has been rejected in two Dutch and one Australian study [37–39]. These clearly showed that different values will be collected with a multi-item VAS for a fixed set of health states if these are presented along with varying other states. In addition, the choice and phrasing of the anchors leads to different results [40].

As such, the measurement procedure of the DC seems to have an advantage over VAS, as the former may be free of certain biases that seem to occur in the VAS. Moreover, DC modeling offers several attractive characteristics not found in other methods of health-state valuation. It is grounded in modern measurement theory, and the judgmental task and the analysis are executed within one unifying framework. In addition, this measurement framework can be extended to other strong measurement models, such as item response theory and structural equation modeling.

Unlike the DC model, the assignment of values to health states by the VAS is not embedded in a strong theoretical measurement framework [30, 41–43]. An axiomatic approach called measurable value functions [44] has been described to deal with VAS-generated data. These are derived from individual responses using algebraic and deterministic axioms. Violations of theoretical predictions or conditions do not usually lead a behavioral scientist to reject the corresponding theory or hypothesis. An error theory would have to be added to these axioms to make them applicable in the behavioral and social sciences. For the VAS, however, such a probabilistic value method has never been presented. Moreover, as mentioned earlier, two pieces of empirical evidence have been brought to bear against the interpretation of VAS as a measurable value function: context bias and end-state aversion.

Given these concerns about VAS, we would suggest a potentially better alternative: to use DC methods based on ordinal responses. Furthermore, several basic (mathematical) assumptions, conditions, and requirements of the DC model warrant closer examination in the context of health-status measurement.
